# Is corona mortis a historical myth? A perspective from a gynecologic oncologist

**DOI:** 10.4274/jtgga.2018.0017

**Published:** 2018-08-06

**Authors:** İlker Selçuk, İlkan Tatar, Ayşegül Fırat, Emre Huri, Tayfun Güngör

**Affiliations:** 1 Department of Gynecologic Oncology, University of Health Sciences, Zekai Tahir Burak Woman’s Health, Health Practice and Research Center, Ankara, Turkey; 2 Department of Anatomy, Hacettepe University School of Medicine, Ankara, Turkey; 3 Department of Urology, Hacettepe University School of Medicine, Ankara, Turkey

To the Editor;

Corona mortis is the vascular anastomosis between the obturator and external iliac or inferior epigastric vessels. It is also known as the ‘crown or circle of death’ because massive bleeding may occur due to an injury.

The obturator artery arises from the internal iliac artery and lies longitudinally to the obturator foramen on the medial part of obturator internus muscle. Anatomically, the corona mortis is on the retro-pubic part of the superior pubic rami lateral to the symphysis pubis, where a pubic artery or vein in this field may arise from the inferior epigastric or external iliac vessels, lie to the obturator foramen, and be damaged during surgical procedures. The incidence of venous corona mortis is between 27% (1) and 100% (2). On the other hand, the incidence of arterial corona mortis is between 14.8% (3) and 36% (4).

The corona mortis may have several anatomic variations. The vascular supply of the pelvis has many connections and variations, as such, the clinical role of the corona mortis in surgical practice is a matter of importance to prevent significant, uncontrolled bleeding for general surgeons, gynecologists, urologists and orthopedic surgeons during femoral hernia operations, urogynecologic operations such as transvaginal tape procedures, pelvic lymphadenectomies or pelvic fracture operations (5).

During procedures with an anterior approach to the pelvis such as hernioplasty, femoral hernia repair or sometimes transvaginal tape operations, the surgeon may not recognize or see the vascular connections on the retro-pubic area, which is on the posterior parts of the surgically exposed field. However, during operations where the surgeon opens the retroperitoneal area such as in pelvic lymph node dissection, the retro-pubic vascular anastomoses are easily seen after a careful and tiny dissection over the external iliac artery below the inguinal ligament. The corona mortis will be noted over the superior pubic ramus, on the medial part of ligamentum teres uteri, where it enters the inguinal canal. The Figure 1 shows the pubic vein below the inguinal ligament on the posterior part of superior pubic rami. This large area of exposure will maintain quick maneuvers during abnormal bleeding to control the hemorrhage. Our clinical practice of 96 pelvic lymphadenectomies showed an incidence of 2.01% (2/96) arterial anastomoses and we had a total of 4 hemorrhages (4.1%) from the pubic vein (venous corona mortis), which were easily controlled. In that manner, the term *corona mortis* is questionable in gynecologic oncology practice. Nevertheless, the amount of bleeding and the ability to control hemorrhage from an arterial corona mortis could not be foreseen.

## Figures and Tables

**Figure 1:**
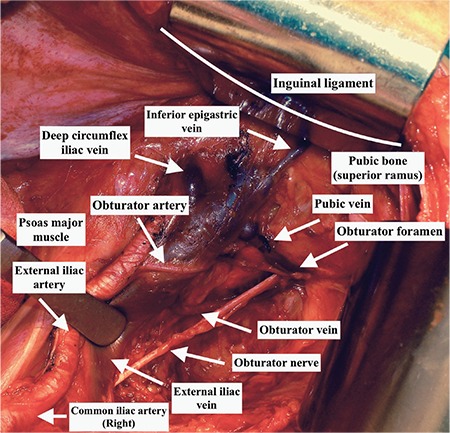
Pubic vein from the obturator vein to the external iliac vein arising from obturator foramen

## References

[1] Lau H, Lee F (2003). A prospective endoscopic study of retropubic vascular anatomy in 121 patients undergoing endoscopic extraperitoneal inguinal hernioplasty. Surg Endosc.

[2] Berberoglu M, Uz A, Ozmen MM, Bozkurt MC, Erkuran C, Taner S, et al (2001). Corona mortis: an anatomic study in seven cadavers and an endoscopic study in 28 patients. Surg Endosc.

[3] Sarikcioglu L, Sindel M, Akyildiz F, Gur S (2003). Anastomotic vessels in the retropubic region: corona mortis. Folia Morphol (Warsz).

[4] Okcu G, Erkan S, Yercan HS, Ozic U (2004). The incidence and location of corona mortis: a study on 75 cadavers. Acta Orthop Scand.

[5] Rusu MC, Cergan R, Motoc AG, Folescu R, Pop E (2010). Anatomical considerations on the corona mortis. Surg Radiol Anat.

